# Clinical and audiological characteristics in adults with tinnitus in South Africa

**DOI:** 10.4102/sajcd.v71i1.1069

**Published:** 2024-11-06

**Authors:** Katijah Khoza-Shangase, Snethemba P. Mkhize

**Affiliations:** 1 Department of Audiology, School of Human and Community Development, University of the Witwatersrand, Braamfontein, South Africa

**Keywords:** tinnitus, audiological characteristics, medical characteristics, adults, retrospective record review, South Africa

## Abstract

**Background:**

In South Africa, the prevalence of tinnitus and its associated clinical and audiological characteristics remain underexplored, posing challenges in the assessment and management of the condition. This knowledge gap affects clinician preparedness and effectiveness in treating tinnitus.

**Objectives:**

This study aimed to explore the clinical and audiological characteristics of adult patients reporting tinnitus at a tertiary academic hospital in South Africa.

**Method:**

A quantitative, non-experimental, retrospective review of 129 patient audiological records from the Audiology Department at Helen Joseph Hospital was conducted. Among these, 71 records were of patients who reported experiences of tinnitus.

**Results:**

Tinnitus was reported by 55% of patients, with a higher prevalence in females (76%). The mean age of patients was 51.9 years. Unilateral tinnitus was present in 28% of cases, while 78% of patients had hearing loss, predominantly sensorineural. Common audiological characteristics included vertigo (24%), otalgia (14%), otorrhea (17%) and perforated tympanic membrane (15%). Clinical conditions associated with tinnitus included hypertension (32%), heart disease (28%), diabetes (9%) and head trauma (9%). Findings regarding age and gender differences in the clinical and audiological manifestations of tinnitus are presented.

**Conclusion:**

Further research with larger, diverse samples and prospective designs is necessary to confirm these findings and explore possible underlying causes.

**Contribution:**

The findings highlight the significant prevalence of tinnitus and its association with various clinical and audiological conditions in the South African context. Understanding these characteristics will enhance the clinician‘s ability to accurately assess, diagnose and manage tinnitus, leading to improved treatment outcomes.

## Introduction

Tinnitus, defined as the conscious perception of sound in the absence of an external acoustic source, is a prevalent and often distressing condition affecting millions globally (De Ridder et al., 2022). Commonly described as ringing, roaring, hissing or humming, tinnitus can be perceived in the ears or within the head (Bauer, 2018). The global prevalence of tinnitus stands at approximately 14%, translating to over 740 million affected adults, with more than 120 million individuals experiencing it severely (Jarach et al., 2022). In South Africa, prevalence estimates among various populations range from 13.51% to 57% (Naude et al., 2020; Ramatsoma & Patrick, 2022; Sidley, 2004).

The aetiology of tinnitus is multifaceted and not fully understood, often linked to underlying conditions, particularly sensorineural hearing loss (SNHL) (Du et al., 2022). Neurological changes in the auditory system can induce hyperactivity in auditory neurons, resulting in tinnitus (Cherian & Kahn, 2011). Chronic tinnitus, a consequence of long-term neuronal changes, leads to permanent alterations in both auditory and non-auditory brain structures (Saeed & Khan, 2021). Somatic tinnitus, another form, is associated with somatic disorders like temporomandibular joint syndrome, whiplash or head injury (Han et al., 2009; Kanji & Khoza-Shangase, 2013).

Tinnitus manifests differently across demographic groups, influenced by factors such as age and gender, and is associated with various clinical and audiological conditions. Among young adults (18–25 years), the prevalence is 9.7%, often linked to loud music exposure (Degeest et al., 2014; Jarach et al., 2022). In the elderly (over 65 years), the prevalence rises to 23.6%, commonly because of presbycusis and medical conditions such as hypertension and diabetes (Jarach et al., 2022; Lasisi et al., 2010).

Tinnitus can be categorised as either objective, a rare form perceived by both the sufferer and an examiner, often linked to vascular or muscular conditions, or subjective, the more common form perceived only by the individual and associated with auditory system lesions (Bauer, 2018; Messina et al., 2022). Whatever its complex aetiology, tinnitus significantly impacts sufferers’ quality of life, potentially leading to concentration issues, depression, anxiety, self-isolation, insomnia and frustration (McCormack et al., 2016).

Assessment of tinnitus is conducted through a multidisciplinary approach, which may include audiological, medical and psychological approaches (Cima et al., 2019), and involves a thorough evaluation of the patient’s medical history, symptoms, lifestyle and possible triggers of tinnitus, supplemented by audiological assessments such as pitch-matching and loudness-matching tests (Yang & Byun, 2016). Because of their expertise and experience in the field, audiologists are an essential part of the team to help patients with tinnitus. The following steps can be facilitated by an audiological treatment for tinnitus: pure tone audiometry, tympanometry and an otoscopic examination incorporating speech tests, interoctaves and high frequencies (Henry et al., 2010). Following an audiological evaluation which includes a thorough case history, psychoacoustic testing should be conducted. This testing includes loudness discomfort levels, tinnitus pitch-matching with octave confusion, loudness-matching minimum masking levels, as well as residual inhibition evaluation. Furthermore, the use of subjective questionnaires should all be included in the basic evaluation of tinnitus (Sweetow et al., 2000). Various questionnaires, including the Tinnitus Handicap Inventory (THI) and Tinnitus Functional Index (TFI), are employed to gauge severity and impact (Cima et al., 2019; Zeman et al., 2012).

Because of tinnitus’ diverse aetiologies, a multidisciplinary approach is often necessary for effective management, incorporating counselling, sound therapy, hearing aids, medication, surgical interventions in the form of cochlear implants, brain stimulation and cognitive behavioural therapy (CBT), in addition to the specific treatments of underlying or co-occurring abnormal contributing factors such as, for example, temporomandibular joint (TMJ) dysfunction (Baguley & Atlas, 2007; Bovo et al., 2011; Han et al., 2009; Henry et al., 2010; Kanji & Khoza-Shangase, 2013; Sweetow & Sabes, 2010; Vielsmeier et al., 2011). Because of the heterogeneity of tinnitus, difficulties in assessing tinnitus, significant placebo effects and poor methodological quality of many treatment trials, the evidence levels for most treatment options are low. A decisive factor in comparisons of treatment efficacy is the requirement for consistency in assessment and outcome measurement (Langguth et al., 2014).

A study conducted in South Africa revealed that audiologists lacked confidence regarding the overall management of tinnitus regardless of their experience in the field (Dawood et al., 2019). There was a lack of training, limited knowledge and no standardised guidelines on tinnitus management (Dawood et al., 2019). The study further highlighted that audiologists have acknowledged the inadequacies in their professional ability to help tinnitus patients and have indicated a need for additional training. Even though most participants offered some kind of counselling to patients, they believed that their knowledge in this field was restricted because fewer audiologists were receiving specialised training to offer counselling to those who had tinnitus (Dawood et al., 2019). Unfortunately, because tinnitus is invisible, resources are not always prioritised, and the lack of resources in South Africa may have an impact on the delivery of tinnitus services (Moroe & Khoza-Shangase, 2014). Participants acknowledged that it can be difficult to provide patients with counselling after they have been treated by doctors who dismiss tinnitus and suggest there is no solution to it (Moroe & Khoza-Shangase, 2014; Sourgen & Ross, 1998). This false belief must be replaced with a constructive one, and it can only occur once audiologists are well-informed about tinnitus and the characteristics associated with the condition (Hoare et al., 2010).

Tinnitus assessment and management are important as they help identify and manage possible causes or triggers of tinnitus. This is crucial as tinnitus has a negative impact on a person’s quality of life; therefore, early identification and appropriate management can help improve a person’s quality of life (Langguth et al., 2014). However, there are several challenges to tinnitus assessment and management. A lack of understanding of the underlying mechanisms can pose a challenge in assessing and managing tinnitus because its underlying causes are not yet fully understood (Dawood et al., 2019). Challenges in tinnitus management arise from its subjective nature and the variability in severity and distress among patients (Dawood et al., 2019). The lack of standardised assessment protocols and limited training and knowledge among audiologists further complicate management (Henry et al., 2010).

Despite the global recognition of tinnitus as a significant auditory and clinical concern, the prevalence and associated characteristics of tinnitus in the South African context remain under-researched. This gap in knowledge poses challenges for clinicians in accurately assessing and managing the condition, as a lack of localised data impedes preparedness and clinical decision-making. Understanding the clinical and audiological characteristics associated with tinnitus in South African patients is crucial for improving diagnostic accuracy and treatment outcomes. This study aimed to address this gap by exploring the clinical and audiological profiles of adult patients reporting tinnitus at a tertiary academic hospital in South Africa.

### Aim

The aim of this study was to explore clinical and audiological characteristics in adult patients who reported experiences of tinnitus in a tertiary academic hospital in South Africa.

### Objectives

- To establish the prevalence of tinnitus in this group of patients.
- To describe the tinnitus characteristics in this group of patients.
- To describe the hearing function in this group of patients (type, degree, laterality and nature of onset).
- To explore the occurrence of audiological characteristics such as ear blockage and/or aural fullness, ear pain, hearing loss, hyperacusis, otorrhea and dizziness.
- To explore the occurrence of medical characteristics such as neck pain, head injury, obesity, hypertension, diabetes and orofacial pain.
- To explore age and gender differences in clinical and audiological manifestations of tinnitus.

## Research methods and design

### Research design

To achieve the outlined objectives of this study, a quantitative non-experimental retrospective record review design was employed (Akhtar et al., 2016; Bloomfield & Fisher, 2019). With this research design, pre-recorded patient records are used to answer research questions and clinical questions (Vassar & Matthew, 2013).

### Description of the site

This study was conducted in the Audiology Department at Helen Joseph Hospital, a tertiary academic hospital comprising 23 wards, 13 clinics and 11 theatres. Because this study aimed to explore clinical and audiological characteristics in adult patients who reported experiences of tinnitus, the data required were extracted from the Audiology Department of the hospital.

Several factors motivated the selection of this particular site. Firstly, Helen Joseph Hospital serves a broad demographic population, encompassing individuals from various socio-economic backgrounds. This diversity allows for a comprehensive exploration of tinnitus experiences across different patient groups, offering insights that are more representative of the wider South African population. The varied clinical presentations encountered at this site ensure that a wide range of clinical and audiological characteristics related to tinnitus can be studied. Secondly, as a tertiary academic hospital, Helen Joseph Hospital handles a high volume of patient referrals, including complex cases that require specialised care. The Audiology Department, in particular, deals with patients presenting with various hearing disorders, including tinnitus, making it an ideal location to study the condition. The large number of patients seen at this facility increases the likelihood of identifying a substantial sample of tinnitus cases, thereby enhancing the reliability and generalisability of the study findings. Thirdly, the hospital maintains detailed and well-organised patient records, which are essential for conducting a retrospective review. These records include valuable information on both the clinical and audiological history of patients, enabling a thorough exploration of the factors associated with tinnitus. Access to such data ensures a rich dataset for analysis, allowing the research to contribute meaningful findings to the current knowledge base on tinnitus in South Africa. Fourthly, Helen Joseph Hospital is affiliated with the University of the Witwatersrand, a leading academic institution in South Africa. This provides an academically stimulating environment, where research is encouraged and supported. The affiliation allows for collaboration with academic professionals and access to research resources, ensuring that the study is conducted with academic rigour and aligns with national research priorities. Lastly, the hospital’s patient population includes individuals who may experience healthcare disparities, reflecting broader health inequalities in South Africa. This provides an opportunity to explore tinnitus in a population that may be underrepresented in global tinnitus research. Moreover, the site allows the study to focus on the unique socio-economic and cultural factors influencing tinnitus experiences and healthcare access in South Africa.

### Sample

A non-probability purposive sampling strategy was used to select participant records (Etikan et al., 2016). The sample size for this study was determined by the number of available records of patients who reported experiences of tinnitus. As per guidelines by Landreneau and Creek (2009), larger sample sizes are generally ideal for robust analysis. In smaller populations, where the sample size (*N*) is 100 or less, it is often preferable to include the entire population in the analysis. For populations around 500, sampling 50% is recommended, and for populations of approximately 1500, a sample of 20% is sufficient. To ensure clinically relevant and statistically meaningful results, at least 10 cases per variable are recommended (Gearing et al., 2006). The sample was drawn from patients who specifically reported tinnitus, ensuring that the sample size was appropriate relative to the total available records. Additionally, the researcher followed the guideline of including at least 10 cases per variable to ensure adequate representation and robust analysis. The researcher also considered practical aspects, such as the time available for data collection and the accessibility of patient records, which ultimately influenced the final sample size of 129 records reviewed, with 71 specifically focussing on patients who reported tinnitus.

The steps followed included: (1) *accessing patient records*, where the researcher gained access to the hospital’s Audiology Department database, which stores records of all patients who have been seen for audiological assessment and treatment; (2) *defining the inclusion criteria*, where the primary inclusion criterion was that the patient’s case history specifically mentioned experiences of tinnitus – here the researcher first reviewed the general pool of patient records to select only those individuals who had audiological evaluations conducted at the hospital; (3) *record selection*, whereby from this general pool, the researcher systematically scanned each case history; flagged patient records if they contained documented evidence of tinnitus complaints, as reported by the patients during their initial consultations; (4) *cross-verification*, where once tinnitus experiences were identified in the case histories, the researcher further cross-referenced these reports with corresponding audiological and clinical data, ensuring that the patients fit the study’s inclusion criteria – any records that lacked detailed information about tinnitus or had incomplete data were excluded from the review and (5) *final sample,* where after careful review and exclusion, the researcher finalised the sample size of 71 records that explicitly documented tinnitus experiences, allowing for a focussed analysis on the clinical and audiological characteristics of those patients. This method ensured a targeted and relevant dataset for exploring tinnitus-related issues in a specific population.

### Inclusion criteria

- Records of patients over the age of 18 years were seen in the department.
- Records of patients who reported experiences of tinnitus in their case history.
- Records of patients seen by the audiologist within the past year (2022).

For this study, 129 patient records of patients who were seen by the audiologist in the past year (2022) were included. Among these, 71 records were of patients who reported experiences of tinnitus.

### Methods of data collection

Following ethical clearance (Protocol number: M230633) and permission from all relevant authorities (Hospital CEO and Audiology Department’s HOD), a pilot study of the self-developed data extraction form was conducted at the University’s Speech and Hearing Clinic to test the research tool and the design of the study. Following amendments to the tool and design based on findings from the pilot study, the main study was conducted.

The researcher went through the adult patient files seen during the past year (2022) at the research site and selected files to be included in the study based on the inclusion criteria. The patient records used for data collection were paper-based and stored in the Audiology Department’s records at Helen Joseph Hospital. These records were retrieved by the researcher, who manually reviewed them in the Audiology Department. The relevant information, including socio-demographic data, medical history, clinical and audiological characteristics, and tinnitus history, was then extracted and recorded electronically using a standardised data-capturing form on an Excel spreadsheet. The entire data collection process took approximately 8 weeks to complete.

To ensure the accuracy and reliability of the data, intra-rater reliability measures were implemented. A random sample of 10% of the extracted records was rechecked to verify consistency in data extraction. However, specific formal reliability and validity measures for the tool were not described, although the pilot testing phase helped mitigate potential issues related to the tool’s reliability. All data relevant to the current study were captured on the self-developed data-capturing form.

The data extraction tool for this study was developed through a structured and systematic process to ensure that it captured relevant, comprehensive and accurate information regarding the clinical and audiological characteristics of patients with tinnitus. The development involved several key steps. Firstly, the initial step in designing the data extraction tool involved a thorough review of existing literature on tinnitus. This review focussed on identifying critical variables related to tinnitus, such as socio-demographic factors, medical history, audiological findings and clinical symptoms. The insights gathered from previous studies helped determine which aspects of tinnitus had been commonly reported and which characteristics were significant for understanding the condition, ensuring that the tool was evidence-based. Secondly, based on the literature review and study objectives, the key variables to be extracted were identified, and these included socio-demographic data, medical history, audiological data and tinnitus-specific data. Thirdly, the tool was developed in consultation with audiologists and other clinical experts from the hospital and university. This step ensured that the tool was aligned with the clinical realities of assessing and managing tinnitus in a tertiary academic hospital. Clinicians provided input on which variables were most relevant for understanding the clinical presentation and management of tinnitus. Then, before full implementation, the data extraction tool was pilot-tested on a small subset of patient records (five). This helped to ensure that the tool was user-friendly, comprehensive and efficient for data capture.

Feedback from this pilot phase allowed for the refinement of the tool, ensuring that it was appropriately structured for large-scale data extraction. After the pilot study was conducted, minor amendments were made to both the data extraction tool and the study design to enhance clarity and ensure comprehensive data capture. Additional fields were added to the data extraction tool to capture more specific clinical and audiological characteristics related to tinnitus (e.g. separate fields for vertigo, otalgia and ear infections), and clarifications were made to some sections of the tool, such as the medical history section, to ensure consistency in how comorbidities like hypertension and diabetes were recorded. After incorporating feedback from the pilot testing and clinical consultations, the tool was finalised.

The final version of the form contained six sections that comprised socio-demographic profile data, medical history data, clinical and audiological characteristics data, as well as tinnitus history data. Socio-demographic profile data captured general background information about the patients such as age and gender. Medical history data recorded any pre-existing or co-occurring medical conditions that might be relevant to the onset or exacerbation of tinnitus, such as hypertension, heart disease, diabetes, dental history, head trauma and other chronic conditions such as thyroid problems, kidney disease or neurological disorders that might be linked to tinnitus. The clinical characteristics data section focussed on the clinical symptoms reported by the patient and identified during examination, including vertigo, otalgia, otorrhea, perforated tympanic membrane, headache and hearing loss. The audiological characteristics data section captured information related to the patient’s hearing profile and ear health such as type, degree and symmetry of hearing loss, previous noise exposure and use of hearing aids. The tinnitus history data section recorded detailed information regarding the patients’ experiences with tinnitus, including onset, duration, frequency, etc. The structure of the tool allowed for systematic and organised data extraction, facilitating ease of analysis and ensuring that no critical information was overlooked. All the data captured from these sections were systematically entered into an Excel spreadsheet.

### Ethical considerations

Throughout the process of the current study, the researcher took ethical principles into consideration in compliance with the 2001 revised Declaration of Helsinki on Research with Human Subjects of 1964 by the World Medical Association (WMA) (Human & Fluss, 2001). In this retrospective data review study, several ethical considerations were considered to ensure the protection of patient rights and confidentiality. Firstly, approval from the relevant institutional ethics review board was obtained prior to the commencement of the study, ensuring that the research adhered to ethical guidelines. Ethical clearance to conduct this study was obtained from the University of the Witwatersrand Human Research Ethics Committee (Medical) with reference number M230633. Secondly, informed consent was not required because of the nature of the retrospective study; however, efforts were made to anonymise patient records to protect their identities and sensitive information. Data were accessed and handled in compliance with hospital policies and relevant legal regulations, such as the *Protection of Personal Information Act* (*POPIA*) in South Africa. Additionally, only aggregated data were reported, ensuring that individual patient information remained confidential. Finally, the research team was trained in ethical research practices to maintain the integrity of the study and uphold the highest ethical standards throughout the data collection and analysis processes.

### Data management

Data management in this study involved systematic procedures for collecting, organising and analysing patient records. Initially, a data extraction tool was developed to capture essential information from the selected patient files. The records, primarily accessed from the hospital’s Audiology Department’s database, were carefully reviewed and entered into an Excel spreadsheet for further analysis. To ensure data accuracy and integrity, the researcher implemented quality control measures, including rechecking a subset of entries to assess intra-rater reliability. This comprehensive approach aimed to maintain high standards of data management, facilitating effective statistical analysis and ultimately contributing to the validity of the study’s findings. Data captured were de-identified for anonymity and confidentiality, and these data were stored in a safe password-protected computer that only the researchers had access to.

### Data analysis

Descriptive statistics were employed to describe the data, and the researcher was able to categorise, summarise and illustrate observations systematically (Pentang & Pentang, 2021). To analyse the gender and age differences in clinical and audiological manifestations of tinnitus, the following inferential statistics were conducted: (1) Chi-square test for unilateral vs. bilateral tinnitus; (2) Chi-square test for medical conditions (hypertension, heart disease) and the (3) *t*-test for the degree of hearing loss (Sullivan-Bolyai & Bova, 2014).

## Results and discussion

The study aimed to explore the clinical and audiological profiles of adult patients reporting tinnitus at a tertiary academic hospital in South Africa. The results will be presented with a discussion that will contextualise the findings within the South African healthcare landscape, highlighting implications for clinical practice and public health initiatives.

### Participants’ profile and prevalence of tinnitus

A sample of 129 participant records was reviewed, and 71 (55%) of these records were of patients who reported experiences of tinnitus in their case history. The gender distribution in the group with tinnitus indicated that the majority were female (*n* = 54, 76%), with males comprising (*n* = 17, 24%). The mean age of these participants was 51.9 years, with ages ranging from 21 to 94 years. The age group with the most occurrences of tinnitus was 60–79 years (*n* = 32, 45%).

The 55% prevalence found in this study is consistent with those found in the study by Naude et al. (2020) in HIV (human immunodeficiency virus)-positive patients in a medical centre in Limpopo, where a prevalence of tinnitus was established to be 57%. Furthermore, the prevalence of tinnitus in this current study was slightly higher than the prevalence established by Ramatsoma and Patrick (2022) (41.5%) in hypertensive patients in a tertiary hospital in Johannesburg. However, the prevalence of tinnitus in this study was inconsistent with that established by Jarach et al. (2022) and McCormack et al. (2016) in a systematic review, which found that the overall global prevalence of tinnitus ranges between 4.1% and 37.2%. These differences in prevalence across studies may be because of variations in tinnitus occurrence and management among the defined populations. The current percentage that is significantly higher in this specific South African sample may be because of contributing factors including the high incidence of noise-induced hearing loss because of occupational and environmental noise, as well as a lack of awareness and protective measures against noise exposure in many South African communities; the high prevalence of conditions requiring treatments with ototoxic medications such as tuberculosis (TB), HIV/acquired immunodeficiency syndrome (AIDS) and cancers, as well as the fact that the current sample had a higher number of participants in the age group where presbycusis is prevalent.

As far as the gender and age distribution were concerned, a higher prevalence of tinnitus among females (76%) compared to males (24%) was found, and the mean age of participants was 51.9 years. This aligns with existing literature suggesting that tinnitus is more commonly reported by older adults, possibly because of presbycusis. The higher female prevalence might reflect a greater tendency among women to seek medical consultation or report auditory symptoms compared to men.

### Describing tinnitus characteristics

**FIGURE 1:**
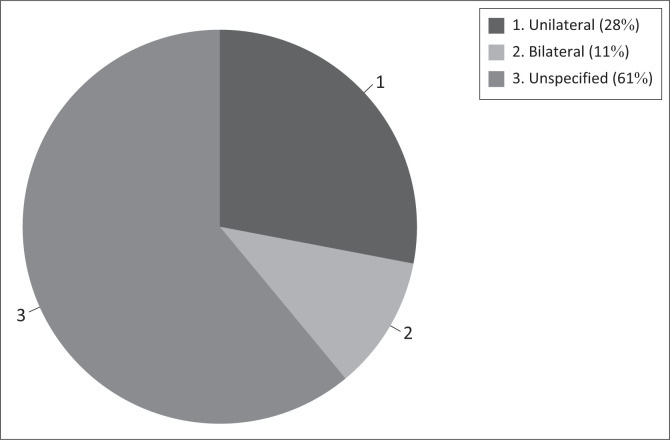
Location of tinnitus recorded.

As depicted in Figure 1, among the participants who reported experiences of tinnitus, 11% experienced tinnitus bilaterally, 28% experienced tinnitus unilaterally (40% in the right ear and 60% in the left ear), and the majority, 61% had no specified location.

Table 1 illustrates the types of tinnitus recorded.

**TABLE 1:** Tinnitus types recorded (*n* = 71, 142 ears).

Type of tinnitus	*n*	%
Pulsatile bilaterally	1	1.4
**Pulsatile unilaterally**		
Left ear	1	1.4
Right ear	1	1.4
Non-pulsatile bilaterally	4	5.6
**Non-pulsatile unilaterally**		
Right ear	1	1.4
Constant bilaterally	1	1.4
**Constant unilaterally**		
Right ear	1	1.4
**Infrequent bilaterally**	1	1.4
**Buzzing bilaterally**	1	1.4
**Not indicated**	59	83

In the vast majority of records (83%), the type of tinnitus was not indicated, with a diversity of other descriptors including pulsatile or non-pulsatile, constant or infrequent and buzzing noted – as depicted in Table 1.

The nature of the tinnitus, unilateral inconsistent pulsatile tinnitus, is inconsistent with that reported by Chamouton and Nakamura (2021) where patients reported having acute, unilateral and intermittent tinnitus. Furthermore, Oiticica and Bittar (2015) discovered complaints of intermittent tinnitus among adult residents of São Paulo, Brazil. Most of the patients presented with unilateral tinnitus that was tonal in character.

Variations found in the current study underscore the complex and subjective nature of tinnitus, making it a challenging condition to diagnose and manage. In the South African context, the diversity in tinnitus characteristics necessitates tailored clinical approaches to address individual patient experiences effectively.

### Describing hearing function

As depicted in Table 2, among participants who reported experiences of tinnitus (*n* = 71, 142 ears), a large majority 78% (101 ears) had hearing loss, where the type of hearing loss was mainly sensorineural in nature as seen in 60% (61 ears), with the degree ranging from mild in 54% (55 ears) to profound in 7% (7 ears), and the configuration of hearing loss being mainly sloping (high frequency) in 88% (89 ears).

**TABLE 2:** Hearing function (*n* = 71, 142 ears).

Factor	Ears (*n*)	%
**Hearing function**		
Normal	28	22
Hearing loss	101	78
**Type of hearing loss**		
Sensorineural	61	60
Mixed	25	25
Conductive	15	15
**Degree of hearing loss**		
Mild	55	54
Moderate	15	15
Moderately severe	15	15
Severe	9	9
Profound	7	7
**Configuration of hearing loss**		
Sloping	89	88
Rising	3	3
Flat	9	9

The noteworthy finding that 78% of ears with tinnitus had hearing loss, with SNHL being the most common type (60%) is consistent with the understanding that tinnitus often coexists with hearing loss, particularly SNHL, which is typically associated with damage to the inner ear hair cells. The prevalence of mixed (25%) and conductive hearing loss (15%) highlights the need for comprehensive audiological assessments in patients reporting tinnitus. Current results present a slightly higher prevalence than those of Carrera et al. (2022) who found that 60% of the patients with tinnitus had hearing loss, primarily bilateral SNHL. Additionally, Al-Swiahb and Park (2016), who also conducted a retrospective record review in Korea to characterise tinnitus patients, the study also found that 59.5% of the participants’ cases had hearing loss.

Additionally, the fact that 60% of the patients with hearing loss had SNHL in this sample, presents a slightly lower prevalence than those of Martines et al. (2010), who established that 74.62% of the patients had SNHL. Nonetheless, the findings support the actual theories that the loss of hair cell activity with the subsequent less effective functioning of the cochlea efferent system represents the most common cause of tinnitus (Norena & Eggermont, 2003).

The majority of hearing losses were mild (54%) with profound hearing loss the least (6.4%). The configuration was predominantly sloping (88%), indicating a gradual decline in hearing sensitivity at higher frequencies, which is characteristic of noise-induced hearing loss, ototoxicity and/or presbycusis.

The mild hearing loss being the most prevalent level of hearing loss in the current sample is consistent with findings from other studies that highlighted that mild hearing loss was the most prevalent degree of hearing loss (Han et al., 2021; Kang et al., 2021). Furthermore, the predominance of tinnitus in patients who had sloping hearing loss (88%) supports Lewis et al. (2020) when they say that patients with high-frequency hearing loss (sloping) are likely to report tinnitus as a primary complaint. Additionally, studies on both human subjects and animal models highlighted that tinnitus perception is associated with rapid declines in hearing sensitivity such as in steeply sloped or notched configurations (Boussaty et al., 2021; Yang et al., 2011). Given South Africa’s industrial and mining sectors, these findings emphasise the importance of occupational health initiatives to prevent hearing loss. Moreover, the use of ototoxic medications to treat highly prevalent conditions such as HIV/AIDS and TB adds to this picture, over and above the fact that the majority of the participants in this sample are potentially of presbycutic age.

### Exploring the occurrence of audiological characteristics

**FIGURE 2:**
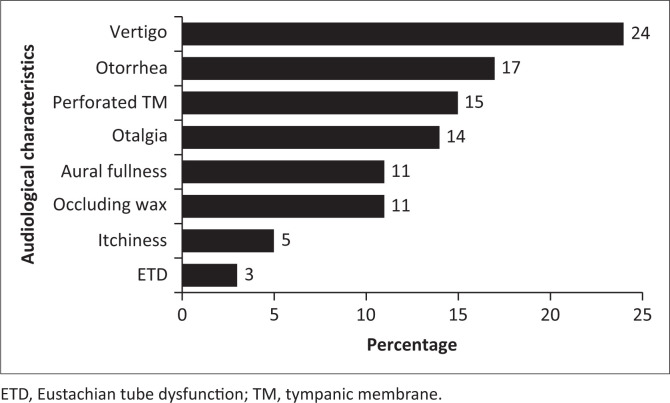
Audiological characteristics recorded.

Figure 2 depicts the audiological characteristics found in the current sample. Among participants who reported experiences of tinnitus, various audiological characteristics were documented. These included vertigo (24%), otorrhea (17%) and otalgia (14%), with Eustachian tube dysfunction (ETD) also documented (3%). The high occurrence of vertigo in this study concurs with Carrera et al. (2022) who established an association between vertigo and tinnitus. Additionally, Al-Swiahb and Park (2016) also found a significant association between the occurrence of tinnitus with hearing loss and vertigo. Moreover, this study found that patients who reported tinnitus in their case history also presented with middle ear pathologies such as otorrhea, otalgia, perforated tympanic membrane, aural fullness and impacted cerumen. These results correlated with Fife and Tourkevich (2021) where it was highlighted that the middle ear or external ear associated with tinnitus included otalgia, effusion and cerumen impaction.

These comorbid conditions can exacerbate the distress caused by tinnitus and complicate its management. In South Africa, where access to specialised audiological services may be limited, these findings underscore the need for primary healthcare providers to be equipped with the skills to manage such complexities.

### Exploring medical characteristics

**FIGURE 3:**
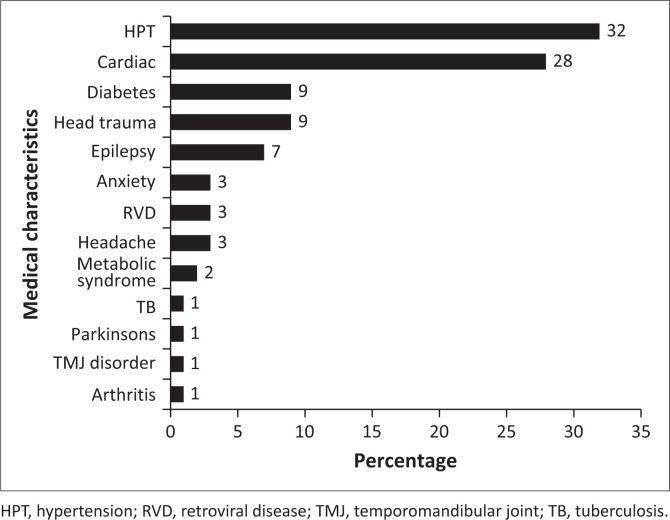
Medical characteristics recorded.

Figure 3 indicates that among participants who reported experiences of tinnitus (*n* = 71), various medical characteristics were reported, with hypertension being the most prevalent condition (32%), followed by cardiac arrest and heart-related diseases (28%), diabetes (9%), head trauma (9%) and epilepsy (7%) with arthritis (1%), Parkinson’s (1%), TMJ disorder (1%), TB (1%) and arthritis (1%) being least reported.

The association between tinnitus and systemic conditions like hypertension and diabetes is well-documented (Lasisi et al., 2010; Sogebi, 2013). The findings in this study correlated with those of Sogebi (2013) and Lasisi et al. (2010), where hypertension was associated with tinnitus. One of the most significant causes of tinnitus onset and persistence is arterial hypertension, which is brought on by widespread microangiopathic alterations that also affect the cochlear artery circulation (Gaspar et al., 2011). A study conducted in South Africa on hypertensive patients also established a 41.5% prevalence of tinnitus in these patients, which further highlights a significant association between tinnitus and hypertension (Ramatsoma & Patrick, 2022). Additionally, the findings on diabetes correlated with those found in a health survey among the adult population in Brazil where it was established that the occurrence of tinnitus was associated with diabetes (Chamouton & Nakamura, 2021). Another clinical characteristic highlighted in this study was head trauma which was consistent with the study conducted in Nigeria (Lasisi et al., 2010) where head injury was significantly correlated with somatosensory tinnitus. These comorbidities suggest that managing tinnitus in South Africa requires a multidisciplinary approach, involving audiologists, general practitioners and specialists such as cardiologists and endocrinologists.

The presence of anxiety (3%) and depression (3%) among the participants aligns with global findings that tinnitus can significantly impact mental health. In South Africa, where mental health services are often under-resourced, there is a critical need to integrate mental health support into audiological care to provide holistic treatment for tinnitus patients.

### Exploring gender and age differences in the clinical and audiological manifestation of tinnitus

#### Gender differences in clinical and audiological manifestations of tinnitus

When a Chi-square test was conducted to examine gender differences in clinical and audiological manifestations of tinnitus as far as gender differences in the prevalence of unilateral vs. bilateral tinnitus were concerned, results showed no statistically significant association between gender and the type of tinnitus (χ^2^(1, *N* = 71) = 2.36, *p* = 0.13), suggesting that the proportion of males and females experiencing unilateral or bilateral tinnitus was not significantly different. For clinical conditions (hypertension and heart disease), there was a statistically significant association between gender and hypertension (χ^2^(1, *N* = 71) = 4.21, *p* = 0.04), with females reporting a higher prevalence of hypertension (40%) compared to males (25%). However, no significant gender differences were found for heart disease (χ^2^(1, *N* = 71) = 0.78, *p* = 0.38) or diabetes (χ^2^(1, *N* = 71) = 1.21, *p* = 0.27). When an independent samples *t*-test was conducted to compare the degree of hearing loss between male and female patients with tinnitus, results indicated that the degree of hearing loss was not significantly different between males (*M* = 45.8 dB, s.d. = 12.6) and females (*M* = 48.2 dB, s.d. = 11.4), *t*(69) = -1.04 and *p* = 0.30.

The findings from this study regarding gender differences in the clinical and audiological manifestations of tinnitus offer nuanced insights into the South African context, aligning with existing literature while highlighting some unique aspects (Basso et al., 2020). Despite the global understanding that tinnitus affects individuals differently based on various factors such as gender (Degeest et al., 2014; Jarach et al., 2022), the results of this study showed no significant association between gender and the prevalence of unilateral or bilateral tinnitus. This finding contrasts with some studies that have suggested gender-based differences in tinnitus presentations, particularly with men reportedly experiencing tinnitus more frequently (Al-Swiahb & Park, 2016; Cederroth & Schlee, 2022). In the present study, both males and females were equally likely to experience unilateral or bilateral tinnitus, suggesting that gender may not play as prominent a role in the type of tinnitus experienced in this population.

A noteworthy finding was the statistically significant association between gender and the prevalence of hypertension, with females exhibiting a higher prevalence (40%) compared to males (25%). This aligns with broader health trends in South Africa, where women are often found to have a higher burden of hypertension (Peltzer & Phaswana-Mafuya, 2013; Sharma et al., 2021). However, no significant gender differences were observed in the prevalence of heart disease or diabetes, mirroring findings from other tinnitus studies where clinical comorbidities often affect both genders similarly.

In terms of audiological outcomes, no significant differences were found between males and females in the degree of hearing loss, which contrasts with research indicating that males typically exhibit greater degrees of hearing loss because of higher exposure to noise-related occupations. This suggests that, within this South African cohort, other factors beyond occupational noise may influence hearing loss severity in tinnitus patients. These results highlight the complexity of tinnitus as a condition and underscore the need for more focussed research on gender-related clinical and audiological variations.

#### Age differences in clinical and audiological manifestations of tinnitus

When a Chi-square test was conducted to examine age differences in clinical and audiological manifestations of tinnitus as far as tinnitus type (unilateral vs. bilateral) was concerned, results showed a statistically significant association between age and the type of tinnitus (χ^2^(1, *N* = 71) = 5.89, *p* = 0.02). Older adults (50 years and above) were more likely to report bilateral tinnitus (40%) compared to younger adults (under 50), who predominantly reported unilateral tinnitus (30%). For clinical conditions, results revealed a significant association between age and the prevalence of heart disease (χ^2^(1, *N* = 71) = 6.22, *p* = 0.01), with older adults reporting higher rates (27%) compared to younger adults (10%). However, no significant differences were found for hypertension or diabetes across age groups. A *t*-test comparing the degree of hearing loss between younger and older adults showed a statistically significant difference (*t*(69) = -2.71, *p* = 0.01). Older adults (*M* = 50.6 dB, s.d. = 11.8) had a significantly greater degree of hearing loss than younger adults (*M* = 42.1 dB, s.d. = 13.2), particularly in the moderate-to-severe hearing loss range.

The findings on age differences in clinical and audiological manifestations of tinnitus highlight key trends in how tinnitus presents differently in older versus younger adults, which aligns with existing literature (Jarach et al., 2022; Lasisi et al., 2010) but also offers some unique insights in the South African context. Results from the Chi-square test showed a statistically significant association between age and the type of tinnitus, with older adults (50 years and above) being more likely to experience bilateral tinnitus (40%), while younger adults under 50 predominantly reported unilateral tinnitus (30%). This supports existing research that suggests bilateral tinnitus is more prevalent in older populations, potentially because of age-related changes in auditory function and increased susceptibility to bilateral SNHL (Al-Swiahb & Park, 2016; Cederroth & Schlee, 2022).

Clinically, the association between age and the prevalence of heart disease was significant, with older adults more likely to report heart disease (27%) compared to younger adults (10%). This finding is consistent with the broader epidemiological trends of cardiovascular conditions, which increase with age and have been linked to tinnitus in older adults because of vascular changes affecting auditory pathways (Jarach et al., 2022; Lasisi et al., 2010). However, no significant differences in the prevalence of hypertension or diabetes across age groups were found, indicating that these conditions may be equally distributed among younger and older adults with tinnitus in this cohort.

In terms of audiological outcomes, older adults exhibited a significantly greater degree of hearing loss than younger adults. The *t*-test results revealed that older adults had an average hearing loss of 50.6 dB compared to 42.1 dB in younger adults, particularly in the moderate-to-severe hearing loss range. This is consistent with presbycusis, or age-related hearing loss, which often exacerbates tinnitus symptoms in older adults (Jarach et al., 2022; Lasisi et al., 2010). These findings underscore the importance of considering age-related factors in the assessment and management of tinnitus, as older adults may require more comprehensive audiological interventions because of the higher prevalence of bilateral tinnitus and greater degrees of hearing loss.

Current findings raise several implications for clinical practice within this context, the key to which is for audiologists to improve their screening, assessment and management of tinnitus patients. Firstly, a multidisciplinary approach to the assessment and management of tinnitus is highlighted. The high prevalence of tinnitus and its association with various audiological and clinical characteristics necessitate a multidisciplinary approach. Collaboration between audiologists, ENT specialists, general practitioners and mental health professionals is essential to address the multifaceted nature of tinnitus. Secondly, given the high prevalence of hearing loss associated with tinnitus, there appears a need for public health initiatives focussing on prevention, early detection and management of hearing loss. Educational campaigns on risk factors such as noise exposure, the importance of hearing protection and regular hearing screenings can help reduce the burden of tinnitus in South Africa. Thirdly, accessibility of services, including mental health services, is raised by current findings. Ensuring access to audiological and mental health services is vital. This includes training primary healthcare providers in tinnitus management and expanding audiology services in rural and underserved areas. Although this implication does not directly emanate from data from the current study, it does require consideration; tele-audiology could also play a significant role in improving access to ear and hearing care. Lastly, patient education and support, where patients are provided with comprehensive information about tinnitus, its causes and management options is crucial. Support groups and counselling services can help patients cope with the psychological impact of tinnitus, improving their overall quality of life.

## Conclusion

This study investigated the prevalence and characteristics of tinnitus among a sample of South African patients, revealing a significant prevalence rate of 55%. The results highlighted the complex nature of tinnitus, its strong association with hearing loss, and its co-occurrence with various audiological and clinical conditions. The high prevalence among females and older adults, the diversity in tinnitus characteristics and the significant proportion of hearing loss, particularly sensorineural, emphasise the need for a comprehensive and nuanced approach to tinnitus management in South Africa. While the study provides valuable insights, several limitations must be acknowledged. The sample size of 129 participants, though adequate for preliminary analysis, may not be representative of the broader South African population. Future studies should aim for larger and more diverse samples to enhance generalisability. The study relied on retrospective data from patient records, which may include incomplete or inaccurate information. Prospective studies with standardised data collection methods could provide more reliable results. Tinnitus characteristics were based on patient self-reports, which can be subjective and influenced by individual perceptions. Objective measures of tinnitus could complement self-reported data for a more comprehensive understanding. The study provides a snapshot of tinnitus prevalence and characteristics without tracking changes over time. Longitudinal studies are needed to understand the progression and long-term impact of tinnitus. While the study explored various audiological and clinical characteristics, it did not extensively analyse potential correlations or causative relationships. Future research should investigate these aspects to elucidate underlying mechanisms. Despite these limitations, the study provides valuable insights that can inform clinical practice, public health initiatives and future research. By addressing these aspects, the burden of tinnitus can be reduced, improving the quality of life for affected individuals in South Africa.
